# 16S rRNA Gene Sequencing Reveals a Shift in the Microbiota of *Diaphorina citri* During the Psyllid Life Cycle

**DOI:** 10.3389/fmicb.2019.01948

**Published:** 2019-08-23

**Authors:** Lixue Meng, Xiaoyang Li, Xiaoqin Cheng, Hongyu Zhang

**Affiliations:** State Key Laboratory of Agricultural Microbiology, Key Laboratory of Horticultural Plant Biology (MOE), College of Plant Science and Technology, Institute of Urban and Horticultural Entomology, Huazhong Agricultural University, Wuhan, China

**Keywords:** Illumina MiSeq sequencing, quantitative real-time PCR, bacterial community, *Diaphorina citri*, developmental stages

## Abstract

The Asian citrus psyllid (*Diaphorina citri*) is a major pest of citrus trees as it transmits *Candidatus* Liberibacter asiaticus (*C*Las). The composition of a host’s microbiota can affect the evolution and ecological distribution of the host. This study monitored the compositional shifts in the citrus psyllid microbiota through all the life stages (egg, nymph 1–5 stages, and adult) by next-generation sequencing (NGS) and quantitative real-time PCR. There were clear differences in both α- and β-diversity of microbiota through the psyllid life stages. Microbiota diversity was markedly higher in the nymph 2–5 stages than in the adult, egg, and nymph 1 stages. Proteobacteria were dominant in all the life stages of *D. citri*, representing >97.5% of the total bacterial community, and *Candidatus* Profftella armature was the dominant genus in all the life stages. Data from the qPCR analysis showed an exponential increase in the populations of three *D. citri* endosymbionts: *Candidatus* Profftella armature, *Candidatus* Carsonella ruddii, and *Wolbachia*. The gut bacterium *Pantoea* was present in all the life stages, but it was markedly higher in the nymph 2–5 stages. The microbiota composition substantially differed among the egg–nymph 1, nymphs 2–5, and adult stages. Therefore, we successfully characterized the microbiota dynamics and thus identified a microbiota shift during the life cycle of *D. citri* by 16S rRNA gene sequencing and quantitative PCR. Moreover, 16S rRNA gene sequencing suggested that *D. citri* acquired the ability to bear *C*Las in the nymph 1 stage. This study enhances our understanding of microbial establishment in the developing *D. citri* and provides a reference resource for the identification of potential biocontrol approaches against this pest.

## Introduction

Recently, microbiotas of insects have attracted great interest, mainly because of the ecological and economical importance of insects (Engel and Moran, 2013; Sugio et al., 2014). In many cases, microbes allow their insect hosts to survive on nutrient-deficient food resources, such as plant sap, conferring a competitive advantage on them (Beck et al., 2018). In addition, arthropod-associated microbes play numerous roles during the life cycle of the host arthropod, including development, reproduction, speciation, immunity, and defense against predators (Oliver et al., 2014; Macke et al., 2017).

The sap-sucking Asian citrus psyllid, *Diaphorina citri* Kuwayama (Hemiptera: Liviidae), is one of the most serious agricultural pests in the citrus-growing regions around the world. It is the vector of *Candidatus* Liberibacter asiaticus (*C*Las). This bacterial species causes the highly destructive huanglongbing (HLB) disease, also called citrus greening, in citrus trees (Grafton-Cardwell et al., 2013). Like many other plant-feeding insects, they rely on bacterial endosymbionts to acquire the amino acids and other nutrients missing in their diet. The obligate bacterial endosymbionts, *Candidatus* Carsonella ruddii, *Candidatus* Profftella armatura, and *Wolbachia* have cooperative and close associations with *D. citri* (Thao et al., 2000; Nachappa et al., 2011; Nakabachi et al., 2013b). The roles of *Carsonella* and *Profftella* in *D. citri*’s life cycle have been deduced by genome sequencing, but *Wolbachia*’s role is still relatively elusive (Saha et al., 2012). *Carsonella* acts as a nutritional symbiont (Tamames et al., 2007; Nakabachi et al., 2013a), and *Profftella* has been predicted to confer a defense advantage because it produces diaphorin, a mildly cytotoxic polyketide (Nakabachi et al., 2013b; Ramsey et al., 2015). Many studies have attempted to explore the endosymbionts, the internal microbiota, of *D. citri*. PCR-RFLP and DGGE methods have been performed to evaluate presence of bacteria in *D. citri* (Subandiyah et al., 2000; Yin et al., 2011). Additionally, the diversity of the cultivable microbiota of *D. citri* has recently been investigated by culture-dependent methods and 16S rDNA clone library (Kolora et al., 2015). Quantitative real-time PCR (qPCR) analyses showed that the *Carsonella*, *Profftella*, and *Wolbachia* levels were positively correlated with each other (Dossi et al., 2014; Chu et al., 2016). Previous studies have provided valuable information regarding the general bacterial diversity and identity of common internal bacteria and potential endosymbionts. However, cloning is a laborious and costly technique with a limited outcome for the detection of scarce bacteria (Zolnik et al., 2016), and many of the early studies were descriptive and confined to adults. There is limited information about the lifelong dynamics and trans-generational transfer of *D. citri*’s microbiota. We used next-generation sequencing (NGS) in our study, given that this technique has been powerful for in-depth compositional and functional analysis of microbiotas and characterization of the relationships between the microbial communities (Hovatter et al., 2011; Costello et al., 2012).

Wild populations of *D. citri* were used in this study. The bacterial community associated to different life stages of *D. citri* (egg, nymph 1–5 stages, and adults) has been analyzed through Illumina MiSeq sequencing. Insects exhibit complex symbiotic interactions with microbes, which have a strong impact on insect physiology and provide resources for developing species-specific pest management tactics (Douglas, 2007; Hamby and Becher, 2016). Understanding microbiota composition and dynamics during *D. citri*’s life cycle will enhance the pest management strategies in the future. From a fundamental perspective, this knowledge is also important to elucidate the role of bacteria in *D. citri*’s life.

## Materials and Methods

### Sample Collection, Processing, and DNA Extraction

*Diaphorina citri* at seven different life stages were collected from a navel orange orchard in Ganzhou city, Jiangxi Province (32°13′ N, 114°08′ E), China. The psyllid eggs were harvested with a sterilized blade, and then pooled together. The adults and nymphs were individually collected with a camel hairbrush. Sexing was not performed during any of the life stages. The psyllid samples were cleaned with successive washes of 3% hydrogen peroxide (1 min vortex), 70% ethanol (twice for 30 s each), and deionized H_2_O (for 2 min) to remove environmental contaminants before DNA extraction. Samples of each life stage (egg, nymph 1–5 stages, and adult) were divided into three pools, with 300 eggs, 200 nymphs 1–3, 100 nymphs 4–5, and 80 adults per pool. Total nucleic acid was extracted using the Qiagen DNeasy Blood & Tissue Kit (QIAGEN, Valencia, CA, United States), according to the manufacturer’s instructions.

### Microbiota Sample Preparation and Sequencing

Separate amplicon libraries were prepared for each psyllid sample according to the Illumina MiSeq 16S Metagenomic Sequencing Library Preparation protocol (Klindworth et al., 2013). Amplicon libraries targeting the V3–V4 hypervariable regions of the 16S rRNA gene were generated (Illumina, San Diego, CA, United States). PCR amplification was performed with primers 338F (5′-ACTCCTACGGGAGGCAGCA-3′) and 806R (5′-GACTACHVGGGTWTCTAAT-3′) under the following thermal cycling conditions: 95°C for 3 min, followed by 25 cycles of 95°C for 30 s, 55°C for 30 s, and 72°C for 30 s, with a final extension at 72°C for 5 min. All psyllid DNA samples were amplified in triplicates and subsequently pooled to minimize PCR bias.

The amplicons were purified using solid phase reversible immobilization (SPRI) beads to remove DNA < 60 base pairs (bp) long (primers and primer dimers), and DNA > 700 bp (Off-target PCR products). The resulting amplicon was approximately 550 bp long. Sequencing was performed with an Illumina MiSeq using the MISEQ REAGENT KIT (version 2; Illumina, Inc., San Diego, CA, United States).

### Statistical Analyses of *D. citri*’s Microbiota From Different Psyllid Life Stages

Illumina FASTQ files were demultiplexed and quality-filtered (q20) with quantitative insights in microbial ecology (QIIME 1.9.1) (Caporaso et al., 2010). Paired-end reads were aligned using FLASH and Trimmomatic, and then assigned to operational taxonomic units (OTUs) using a 97% pairwise identity threshold. The Greengenes taxonomic database was used with an open reference OTU picking strategy for taxonomic assignment^1^.

For the analysis of the psyllid life stages, the sequence data were rarefied to a depth of 12,141 sequences. The results were plotted on a rarefaction curve, and analysis of variance (ANOVA)-Tukey’s test was performed to assess the differences between the psyllid life stages. The microbial alpha diversity indices were estimated to uncover the bacterial diversity (Simpson and Shannon) and species richness (Chao1 and ACE). The microbial beta diversity was estimated using weighted and unweighted UniFrac analyses to compare different samples. The adonis function, that performs a permutational multivariate analysis of variance (PERMANOVA) with 999 permutations was used to determine the percent variation explained by developmental stages, as well as the significance and effect size for both weighted and unweighted UniFrac distance matrices. Principal coordinate analysis (PCoA) was used to compare differences among psyllid samples based on both UniFrac distance matrices.

### Quantitative Real-Time PCR (qPCR)

Both the forward and reverse primers (338F and 806R) used for the 16S rRNA gene sequencing contained multiple mismatches to *Carsonella* genome, biasing the amplification against *Carsonella*’s 16S rRNA gene (Morrow et al., 2017). To quantify *Carsonella* and determine the density of the other three known symbionts (*Profftella*, *Wolbachia*, and *C*Las) at different stages of *D. citri*, we conducted an absolute qPCR analysis as described previously (Chu et al., 2016).

Both TaqMan (wingless, *C*Las) and SYBR green methods (*Carsonella*, *Profftella*, and *Wolbachia*) were used in this study (Table 1). The wingless host gene established by Li et al. (2008) was selected as endogenous control. Standard curves were generated to ensure that quantification of the same target gene on different qPCR plates was comparable. The qPCR assays were conducted in triplicates with a CFX384 Touch Real-time PCR cycler (Bio-Rad, Hercules, CA, United States). Each reaction mixture was composed of PCR Master Mix (Roche), 0.4 μL of each primer (10 μmol/L), and 1 μL of DNA template (15 ng) in a final volume of 10 μL. The bacterial concentration in each sample was calculated relative to the Ct values in the standard curves, which were generated by using 10-fold serial dilutions of specific DNA fragments.

**TABLE 1:** Primers and probes used for the qPCR assays.

**Target species**	**Target gene**	**Assay type**	**Primer/probe sequence**	**References**
*C*Las	16S rDNA	TaqMan	5′-TCGAGCGCGTATGCGAATAC-3′ 5′-GCGTTATCCCGTAGAAAAAGGTAG-3′ 5′ -AGACGGGTGAGTAACGCG- 3′	Li et al., 2006; Coy et al., 2014
*D. citri*	wingless	TaqMan	5′-GCTCTCAAAGATCGGTTTGACGG-3r 5′-GCTGCCACGAACGTTACCTTC-3′ 5′-TTACTGACCATCACTCTGGACGC-3′	Li et al., 2008
*Carsonella*	16S rDNA	SYBR	5′-TGGGAACGCCATATGCTAAT-3′ 5′-GTCCCAATGGGTTGTTCATC-3′	Dossi et al., 2014
*Profftella*	16S rDNA	SYBR	5′-GCCTTTATGGGTAGGGCTTC-3′ 5′-CCGGACTACGATGCACTTTT-3′	Dossi et al., 2014
*Wolbachia*	*ftsZ*	SYBR	5′-AGCAGCCAGAGAAGCAAGAG-3′ 5′-TACGTCGCACACCTTCAAAA-3′	Dossi et al., 2014

For each sample, the copy numbers of the target genes in the template DNA were calculated as described by Whelan et al. (2003). Subsequently, the symbiont copy number was divided by the wingless gene copy number in the same sample. The *C*Las density of the samples was compared by dividing infected *D. citri* into high- and low-infection groups using a density threshold of 0.02 copies of *C*Las 16S rDNA per copy of wingless gene (Chu et al., 2016). Statistical analyses (chi-square test of homogeneity and ANOVA) were conducted on the symbiont densities. To satisfy the condition of normality for ANOVA, symbiont densities were transformed for statistical analysis using log (1 + x) (*Wolbachia*) or log (*Carsonella* and *Profftella*) as previously described (Chu et al., 2016).

## Results

A total of 21 samples, with three pools for each category (egg, nymph 1–5 stages, and adult) generated 943,384 raw reads in one Illumina MiSeq flow cell. After filtering, 820,407 sequences were assigned taxonomy. This resulted in 31,174–44,938 high-quality merged sequences per sample (Table 2). On average, the conditional uncovered probability of the samples indicated a high likelihood that over 99% of the bacterial taxa in the samples were covered. Rarefaction curves from a depth of 1,000–100,000 sequences indicated enough sequencing coverage as demonstrated by the observation that the OTU accumulation curves reached a plateau (Supplementary Figure S1).

**TABLE 2:** Summary of the 16S rRNA read counts for all *D. citri* samples.

**Sample ID**	**Number of reads**	**Total bases**	**Min length (bp)**	**Max length (bp)**
Egg-1	30348	13562469	230	470
Egg-2	41152	18380901	363	500
Egg-3	41159	18405385	421	504
Nymph1-1	43703	19402335	272	487
Nymph1-2	31286	14029091	421	481
Nymph1-3	40738	18270588	419	483
Nymph2-1	33999	15228671	298	498
Nymph2-2	31926	14337403	377	507
Nymph2-3	31466	14141846	318	453
Nymph3-1	40087	18005174	398	505
Nymph3-2	44595	19991297	308	457
Nymph3-3	43473	19497182	270	468
Nymph4-1	44938	20150894	421	499
Nymph4-2	31174	14001547	338	504
Nymph4-3	42101	18863849	283	476
Nymph5-1	38953	17401716	311	502
Nymph5-2	43608	19563038	363	468
Nymph5-3	42460	19018686	339	499
Adult-1	38828	17411750	377	453
Adult-2	42068	18731831	422	504
Adult-3	42345	18853781	317	504

### 16S rRNA Gene Sequencing Data

In total, 451 distinct OTUs were clustered across the seven life stages. The alpha diversity indices were estimated to determine the bacterial diversity (Simpson and Shannon indices) and species richness (Chao1 and ACE indices) (Table 3). Across the various life stages, there were significant differences in the bacterial diversity estimated by Simpson and Shannon indices (ANOVA, *F* = 11.078, *df* = 4, 20, *p* < 0.001; Table 3), and as *D. citri* matured from nymph to adult, the bacterial diversity showed a declining trend. The bacterial diversity of nymphs 2–5 was significantly higher than that of adult (*p* < 0.001), egg (*p* < 0.01), and nymph 1 (*p* < 0.01), but the bacterial diversity did not significantly differ among the egg, nymph 1, or adult stages (*p* > 0.15). Analysis of species richness estimated by Chao1 and ACE indices indicated differences among the life stages, but this difference did not reach a significant level (ANOVA, *F* = 2.025, *df* = 4, 20, *p* = 0.13; Table 3). The average species richness of nymphs 2–5 was higher than that of adult (*p* > 0.10), egg (*p* > 0.10), and nymph 1 (*p* > 0.10).

**TABLE 3:** Estimation of species richness and diversity of *D. citri* microbiota during psyllid life stages.

**Sample ID**	**Bacterial diversity**		**Species richness**	
		
	**Shannon**	**Simpson**	**Ace**	**Chao**
Egg	0.60 ± 0.03	0.75 ± 0.02	21.73 ± 0.61	20.75 ± 0.71
Nymph 1	0.51 ± 0.24	0.73 ± 0.13	22.88 ± 4.49	23.28 ± 7.30
Nymph 2	0.98 ± 0.04	0.51 ± 0.06	31.82 ± 3.69	31.08 ± 3.17
Nymph 3	0.93 ± 0.07	0.55 ± 0.07	32.19 ± 5.13	31.39 ± 5.76
Nymph 4	0.91 ± 0.13	0.55 ± 0.10	32.16 ± 1.28	32.00 ± 2.71
Nymph 5	1.07 ± 0.15	0.44 ± 0.02	32.75 ± 6.29	31.00 ± 1.80
Adult	0.37 ± 0.01	0.86 ± 0.00	36.06 ± 12.09	33.10 ± 11.17

### Community Characterization

**FIGURE 1:**
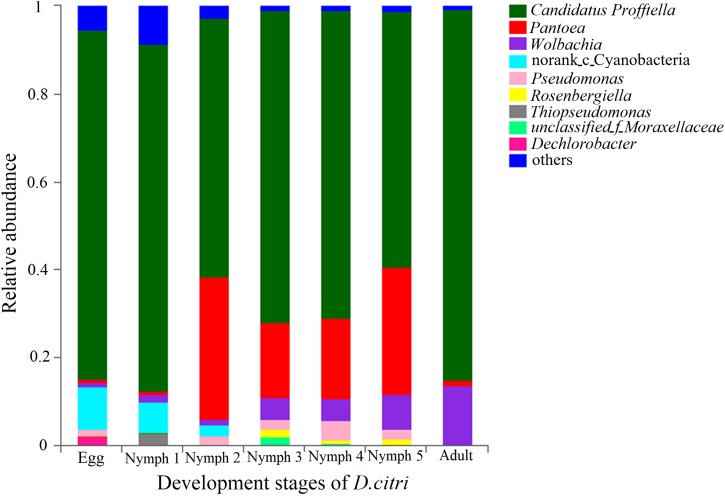
Relative abundances of the bacterial genera in the pooled psyllid samples from the seven developmental stages. Only taxa with a relative abundance ≥1% in at least one sample were analyzed.

The phylum with the highest relative abundance (97.5% of all sequences) across all the developmental stages was found to be the Proteobacteria. The remaining sequences were classified into Cyanobacteria, Actinobacteria, Firmicutes, Bacteroidetes, and Acidobacteria. Among the Proteobacteria, the dominant class was β-Proteobacteria (73.0% of all Proteobacteria), followed by γ-Proteobacteria (18.7%), and α-Proteobacteria (5.83%). Cyanobacteria were only present during the egg, nymph 1 and nymph 2 stages with a decreasing abundance as *D. citri* developed (from 11.8 to 0.03%). *Profftella* was clearly the dominant genus (mean = 58.1–84%) during all the stages and seven genera (along with one family-level taxon and one Norank-c-Cyanobacteria) showed a relative abundance of 1% or greater during at least one stage (Figure 1). The adults had the highest proportion of *Profftella* (84%), and thus had fewer genera with a relative abundance ≥1%. *Wolbachia* was the second most abundant genus in the adults (13.6%) and was detected in the samples from all other life stages (>1.1%). *Pantoea* was widely present during all the stages of *D. citri* but was more abundant during the nymph 2–5 stages (>17%). *Thiopseudomonas* and *Dechlorobacter* were only found in egg and nymph 1 samples. *Rosenbergiella* was only detected in the samples from nymph 2–5 stages (Figure 1).

**FIGURE 2:**
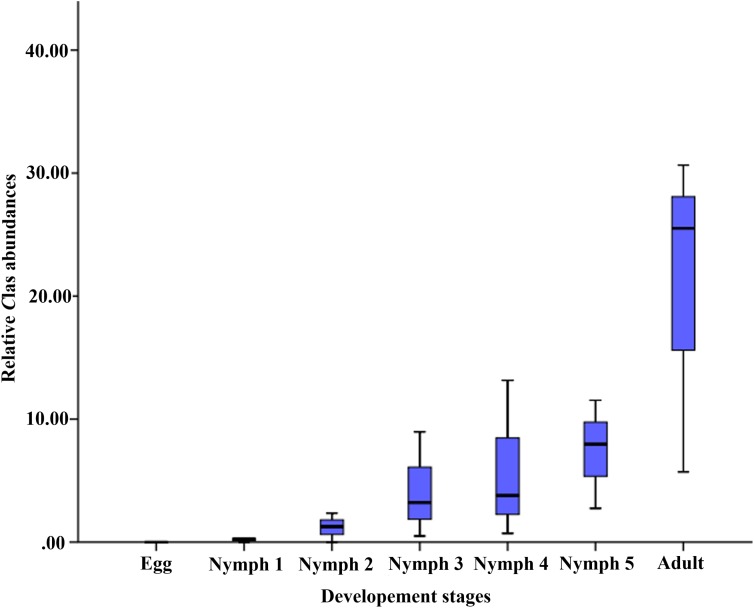
Relative *C*Las abundances (on the y-axis as *C*Las ‱ of all 16S rRNA sequences in samples of the corresponding life stage).

The average relative abundance of *C*Las across the seven life stages was 0‱ (egg), 0.25‱ (nymph 1), 1.25‱ (nymph 2), 3.22‱ (nymph 3), 3.78‱ (nymph 4), 7.96‱ (nymph 5), and 25.51‱ (adult), increasing with age (Figure 2).

### Taxonomic Composition of *D. citri*’s Microbiota as Identified by 16S rRNA Gene Sequencing

**FIGURE 3:**
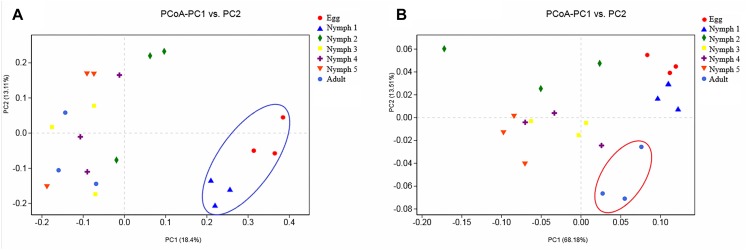
Principal coordinate analysis of the β-diversity values during the seven developmental stages. **(A)** Abundance was ignored in the unweighted UniFrac distances; **(B)** but considered in the weighted UniFrac distances. Confidence ellipsoids around the samples indicate the degree of variation.

Bacterial communities across the seven life stages were significantly different according to a PERMANOVA test (unweighted *F* = 1.47, *R*^2^ = 0.42, *p* = 0.006; weighted *F* = 5.36, *R*^2^ = 0.73, *p* = 0.001). Pairwise PERMANOVA comparisons (Table 4) revealed differences among egg, nymphs and adults’ samples except for comparisons of egg and nymph1 (*p* = 0.6012). Figure 3 illustrated the ordination by PCoA of the Illumina sequencing profiles. The unweighted UniFrac (which does not account for abundance data) PCoA explained 18.4% (axis 1) and 13.11% (axis 2) of the variation across the life stages, with the egg and nymph 1 samples clustering together (*p* = 0.6012) (Figure 3A). On the other hand, the weighted UniFrac PCoA explained 68.18% (axis 1) and 13.51% (axis 2) of the variation, showing that samples from the adults did not cluster with those of earlier stages (*p* < 0.05) (Figure 3B).

**TABLE 4:** Pairwise PERMANOVA comparisons of OTU diversity for different life stages.

**Pairwise PERMANOVA test**	***p* (perm) value**
Egg × Nymph1	0.6012
Egg × Nymph2	**0.0183**
Egg × Nymph3	**0.0198**
Egg × Nymph4	**0.0131**
Egg × Nymph5	**0.0104**
Egg × Adult	**0.0024**
Nymph1 × Nymph2	**0.0161**
Nymph1 × Nymph3	**0.0154**
Nymph1 × Nymph4	**0.0117**
Nymph1 × Nymph5	**0.0231**
Nymph1 × Adult	**0.0037**
Nymph2 × Nymph3	0.4701
Nymph2 × Nymph4	0.4927
Nymph2 × Nymph5	0.5119
Nymph2 × Adult	**0.0032**
Nymph3 × Nymph4	0.4619
Nymph3 × Nymph5	0.5432
Nymph3 × Adult	**0.0020**
Nymph4 × Nymph5	0.5717
Nymph4 × Adult	**0.0017**
Nymph5 × Adult	**0.0014**

*Bold text indicates significance at p < 0.05.*

### Population Shift in the Abundant Bacteria Species Across the Life Stages as Shown by qPCR

**FIGURE 4:**
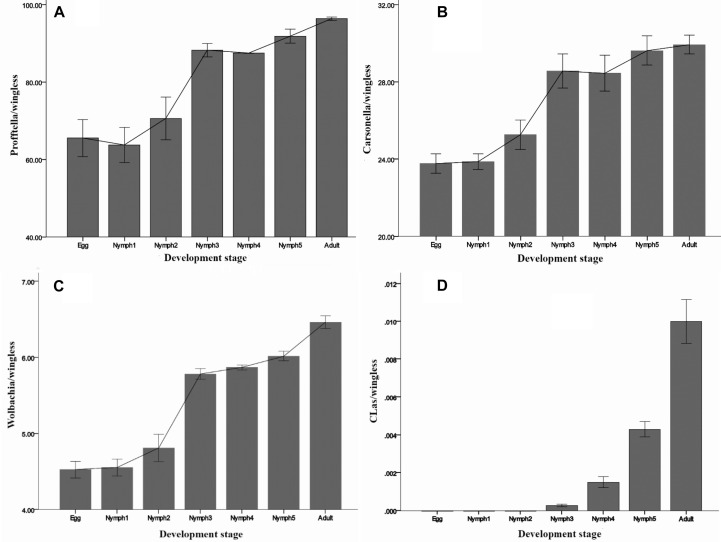
Densities of **(A)** *Profftella*, **(B)** *Carsonella*, **(C)** *Wolbachia*, and **(D)** *C*Las in *D. citri* across the seven life stages. The error bars indicate the standard errors.

By 16S rRNA gene sequencing, *Carsonella* sequence reads in *D. citri* individuals ranged between 0 and 0.2%. However, in the qPCR experiment, the presence of *Carsonella* was confirmed in all individuals, and the content was high (Figure 4B). All the three investigated endosymbionts of *D. citri* showed exponential increases from egg to adult stage (Figure 4). *Profftella*, the secondary endosymbiont, was the dominant genus during *D. citri* development (Figure 4A). Even though *Carsonella* and *Wolbachia* showed trends like *Profftella*, these two species were not as abundant (Figures 4B,C).

*C*Las was detected during the nymph 3–5 and adult stages, but at a low level (<0.02 copies). Inconsistent with the MiSeq sequencing results, *C*Las was not detected in the egg and nymph 1–2 samples by qPCR (Figure 4D). Taken together, these results suggest that in this case qPCR has limitations even though it is a highly sensitive technique. For some low-abundance templates, the adequate amplification for signal detection above the fluorescent background noise may not occur, resulting in false-negatives. NGS due to its high-throughput can detect large amounts of information about the total and active members of bacterial communities at the gene sequence level and can quantify low-abundance species (0–5%) (Webster et al., 2010; van Elsas and Boersma, 2011). Quigley et al. (2014) evaluated the ability of NGS to quantitatively analyze samples with known densities of *Symbiodinium* types and demonstrated that NGS is more sensitive and quantitative than qPCR. Likely this also happened in our case, but further investigations are needed to elucidate the level of *C*Las detection.

## Discussion

A complex microbiota is the basis for the normal survival and development of insects from fertilized eggs to adults (Dillon and Dillon, 2004; Duguma et al., 2013). Here we reported the patterns in microbiota diversity across all the developmental stages of *D. citri*, the primary vector of HLB. RFLP analysis (Subandiyah et al., 2000), qPCR (Dossi et al., 2014; Hussain et al., 2017; Mann et al., 2018), and the Roche 454 pyrosequencing technology (Kolora et al., 2015) have been used in previous studies on psylla microbiotas. Previous studies have provided valuable information regarding the general bacterial diversity and identity of common internal bacteria and potential endosymbionts. However, many of the early studies were descriptive and confined to adults. There is limited information about the lifelong dynamics of *D. citri*’s microbiota.

As in many other insects, *D. citri* microbiota was mainly dominated by species of the Proteobacteria phylum. Diversity estimation revealed that the bacterial communities of *D. citri* significantly differed across the psyllid life stages. The diversity indices of the bacterial communities (Shannon index: 0.37–0.98, Simpson index: 0.44–0.86) were significantly lower than those reported in drosophila flies (Shannon index: 1.99, Simpson index: 0.70) (Wong et al., 2013). Among the herbivore insects examined to date, sap-feeders, such as psyllids, aphids, and whiteflies show the lowest microbial diversity (Shannon’s index: 0.40–1.46, Simpson’s index: 0.15–0.84). Phloem sap’s low bacterial content and simple biological processes may contribute to the low bacterial diversity in sap-feeders (Jing et al., 2014).

Microbiomes display temporal and spatial variability across host developmental stages (Shade and Handelsman, 2012). The beta diversity revealed by PERMANOVA test indicates that the microbiota of *D. citri* differed across the life stages (egg, nymph 1–5 stages, and adult). The microbiotas of egg and nymph 1 were highly different from those of subsequent stages, with a much lower alpha diversity and unique microbial composition dominated by *Profftella* and Norank-c-Cyanobacteria. The microbiota of nymph 1 more closely resembles that of the egg, possibly because psyllids in the nymph 1 stage have just hatched from their eggs and have little contact with outside microbes. Some of these bacteria species, such as members of the genus *Profftella*, are vertically acquired through transovarial transmission in *D. citri*. The photosynthetic bacteria, Cyanobacteria, are not found in the adults, and thus may be acquired from breeding sites (Thiery et al., 1991). One bacterial group that appears to be particularly important in the nymph 2–5 stages is *Pantoea*, and its abundance is significantly higher than that in other stages. *Pantoea* are ubiquitous plant pathogens but they have also been described as part of the gut microbiota in *D. citri* and some aphid species (Engel and Moran, 2013). Some taxa of *Pantoea* have been known to degrade and utilize different types of plant materials (Morales-Jiménez et al., 2009). The recruitment of *Pantoea* in nymphs 2–5 may play a key role in the initiation of digesting external nutrient sources. However, its dominance in *D. citri* was transient. In the adult stage, *Profftella* and *Wolbachia* are the most abundant genera in *D. citri. Wolbachia* has been shown to spread throughout the insect’s somatic tissues and germline (Dobson et al., 1999). Increased abundance of *Wolbachia* is thought to be associated with organ development (Dobson et al., 1999; Ramsey et al., 2017). The effects of developmental stages on the microbiota of other herbivore insects were also reported. In the sweet potato whitefly (*Bemisia tabaci*), the bacteria diversity was the highest in the nymph stage and then decreased at maturity stages (Indiragandhi et al., 2010), while in the desert locusts (*Schistocerca gregaria*), there was often a successional increase in diversity with age (Dillon et al., 2010).

Besides providing a general insight into the microbiota of *D. citri*, our study also analyzed the dynamics of the known endosymbionts. Psyllids have a specialized organ called bacteriome, where coexisting microbes have developed intimate interactions (Spaulding and von Dohlen, 1998). *D. citri’*s bacteriome harbors bacterial endosymbionts *Carsonella*, *Profftella*, and *Wolbachia*. The primary endosymbiont *Carsonella* resides in psyllid bacteriocytes in the external cell layer of the bacteriome, whereas *Profftella* is found in the syncytial cytoplasm within the interior of the bacteriome (Nakabachi et al., 2013b; Hosseinzadeh et al., 2019). Both bacteria are vertically transmitted from mother to offspring (Hosseinzadeh et al., 2019). *Wolbachia* is also localized to the outside layer of the bacteriome, as is the case with *Carsonella* (Hosseinzadeh et al., 2019). Unlike the bacteriocyte-limited endosymbionts *Profftella* and *Carsonella*, *Wolbachia* is scattered throughout the *D. citri* body and has the highest density in the Malpighian tubules (Hosseinzadeh et al., 2019). *Wolbachia* is predominantly transmitted maternally, but also occasionally horizontally between species (Werren et al., 2008; Zug et al., 2012). We identified that the infection titers of the three endosymbionts tended to increase with successive psyllid life stages. The exponential growth of the three endosymbionts across the stages of *D. citri* is synchronized with the organogenesis of the bacteriome as previously observed under microscopy (Dossi, 2013). Simonet et al. (2016) suggested that the rapid increase in the numbers of both endosymbionts and bacteriocytes during insect development was linked to the insect’s high nutritional demands given that the nutritional needs of the insect increase as it develops. The metabolic specialization between symbionts and *D. citri* indicates mutually indispensable associations, suggesting that these symbionts may be potential targets for vector control. Furthermore, the adaptative changes in the host’s immune response against the endosymbionts may also explain the changes in symbiotic density during host development (Nishikori et al., 2009; Douglas et al., 2010). Continuous multiplication of the symbionts may be due to their tolerance to or escape from the host immune responses (Welchman et al., 2009). Nevertheless, further research is needed to elucidate the growth dynamics of the endosymbionts in detail (Login and Heddi, 2013; Ramsey et al., 2017).

*C*Las has previously been shown to colonize *D. citri* starting from the nymph 2 stage (Hung et al., 2004). These results were obtained by PCR and qPCR assays. qPCR is a powerful culture-independent method used to quantity microbial fractions or organisms, but limitations exist in terms of resolution. Very low DNA concentrations as well as the presence of inhibitors and contaminants may result in false negative results (Gryndler et al., 2013; Uyaguari-Diaz et al., 2016). Here we used MiSeq sequencing method and found that *D. citri* could also acquire *C*Las in the nymph 1 stage. In our study, *D. citri* eggs were *C*Las-negative, which was in line with previous reports (Grafton-Cardwell et al., 2013; Grafton-Cardwell and Daugherty, 2013).

Overall, in this study we showed that bacterial colonization of *D. citri* is an active and continuous process and the composition of the bacterial community varies throughout the psyllid life cycle. Our findings demonstrated the plasticity of symbiont density in response to host development. Additionally, we showed that *C*Las-acquisition ability of *D. citri* could start as early as nymph 1 stage. Further studies are required to determine whether these strong differences in microbiota diversity and composition across the life stages of *D. citri* may affect its *C*Las transmission. The major limitation of our study is that a single psyllid population from a single citrus field was used. Recent studies have shown that differences in the genetic backgrounds of *D. citri* populations can affect *C*Las and endosymbiont densities (Chu et al., 2016; Ammar et al., 2018). It remains to be elusive whether such factors also influence microbiota dynamics, but worth further investigation.

## Data Availability

All the bacterial 16S rRNA amplicon and metagenomic sequencing data have been deposited in NCBI’s Short Read Archive under the accession number SRP178960.

## Author Contributions

LM and HZ designed the study, conducted the data analysis, and wrote the manuscript. LM performed the fieldwork and the laboratory and data analyses. XL and XC assisted in collecting the research data. All authors contributed to the manuscript revision, and read and approved the submitted version.

## Conflict of Interest Statement

The authors declare that the research was conducted in the absence of any commercial or financial relationships that could be construed as a potential conflict of interest.

## Footnotes

1 http://greengenes.lbl.gov

## Supplementary Material

FIGURE S1 Rarefaction curves of observed OTUs (97% threshold) versus the number of the sampled reads in *D. citri* samples.
