# Supplementary figures and images for: 16S rRNA Gene Sequencing Reveals a Shift in the Microbiota of Diaphorina citri During the Psyllid Life Cycle
Source: Front Microbiol. 2019 Aug 23;10:1948. doi: 10.3389/fmicb.2019.01948 (PMC6716071; doi:10.3389/fmicb.2019.01948)

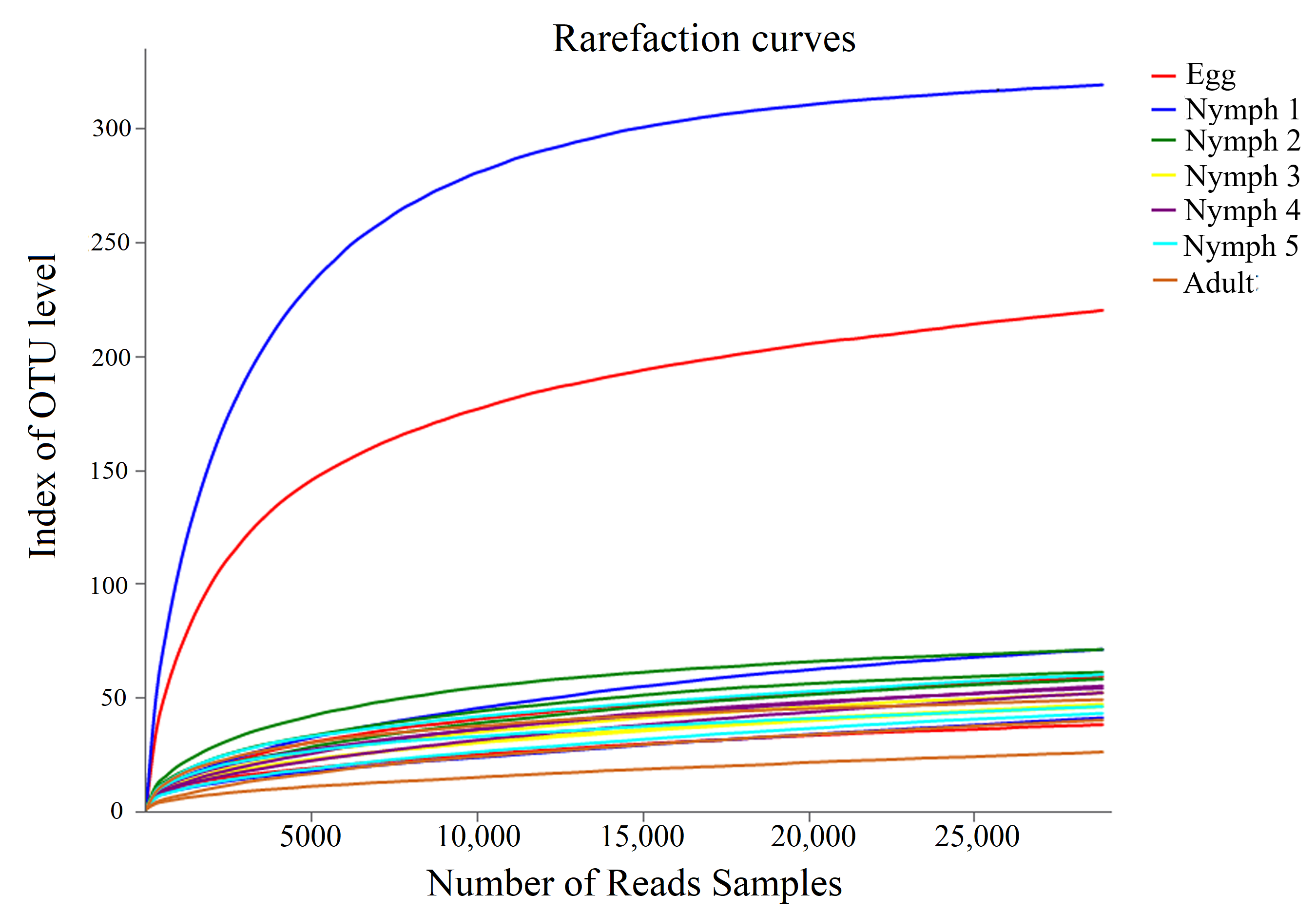

Supplement: FIGURE S1 — Rarefaction curves of observed OTUs (97% threshold) versus the number of the sampled reads in D. citri samples. [file Image_1.TIF]
